# Safety and effectiveness of the first contact force ablation catheter with a flexible tip

**DOI:** 10.1016/j.hroo.2023.10.006

**Published:** 2023-10-31

**Authors:** Devi Nair, Martin Martinek, B. Judson Colley, Sri Sundaram, Ramesh Hariharan, Gustavo Morales, Philipp Sommer, Stewart Healy, Usman Siddiqui, Douglas Gibson, Kristina Chapman, Anne Sarver, Monica Lo

**Affiliations:** ∗ St. Bernard’s Medical Center, Jonesboro, Arkansas; † Cardiology, Angiology, and Intensive Care Medicine, Ordensklinikum Linz Elisabethinen, Linz, Austria; ‡ Jackson Heart Clinic, Jackson, Mississippi; § South Denver Cardiology Associates PC, Denver, Colorado; || University of Texas Health Science Center at Houston, Houston, Texas; ¶ Affinity Cardiovascular Specialists, Birmingham, Alabama; ∗∗ Clinic for Electrophysiology, Herz- und Diabeteszentrum NRW, Bad Oeynhausen, Germany; †† Monash Medical Centre, Melbourne, Australia; ‡‡ Advent Health Orlando, Orlando, Florida; §§ Scripps Health, San Diego, California; |||| Abbott Laboratories, Abbott Park, Illinois; ¶¶ Arkanasas Heart Hospital, Little Rock, Arkansas

**Keywords:** Atrial fibrillation, Atrial flutter, Radiofrequency catheter ablation, Clinical trial, Contact force

## Abstract

**Background:**

Catheter ablation is an established therapy for paroxysmal atrial fibrillation (PAF). The TactiFlex Ablation Catheter, Sensor Enabled (TactiFlex SE) is a next-generation radiofrequency ablation catheter incorporating fiber optics-based contact force–sensing technology with a flexible, laser-cut tip.

**Objective:**

The study sought to evaluate the safety and effectiveness of the TactiFlex SE ablation catheter for treatment of drug-refractory PAF.

**Methods:**

The TactiFlex AF investigational device exemption was a prospective, nonrandomized, multicenter clinical study. Enrollment began on June 26, 2020 and completed June 18, 2021. Subjects with PAF underwent de novo pulmonary vein isolation and, if indicated, ablation for typical atrial flutter. Subjects were followed for 12 months.

**Results:**

Of the 355 subjects enrolled at 37 sites worldwide, 334 underwent ablation with the TactiFlex SE catheter. The Kaplan-Meier estimate of 12-month freedom from AF/atrial flutter (AFL)/atrial tachycardia recurrence was 72.9% (95% confidence interval [CI] 95% CI 67.2%–77.8%) and clinical success was 83.6% (95% CI 95% CI 78.1%–87.2%). As-treated analyses compared subjects treated at high power (left atrium time-averaged power setting 40–50 W; n = 222) vs low power (<40 W; n = 97). The Kaplan-Meier estimate of 12-month freedom from AF/AFL/atrial tachycardia recurrence was 76.4% (95% CI 69.3%–82.0%) and clinical success was 83.9% (95% CI 77.5%–88.6%) in the high-power group compared with 66.8% (95% CI 56.1%–75.5%) and 80.7% (95% CI 70.8%– 87.5%), respectively, in the low-power group. The primary safety event rate in all treated subjects was 4.3%; 4.1% in the HP group and 5.2% in the LP group (*P* = .7671).

**Conclusion:**

TactiFlex SE is safe and effective for treatment of drug-refractory PAF and concomitant AFL and enables more efficient procedures than previous generation catheters.

**Key Findings**

▪ The TactiFlex SE is safe and effective for treatment of drug-refractory paroxysmal atrial fibrillation.
▪ Ablation procedures employing high power (40–50 W) were more efficient, used less fluoroscopy, and trended toward higher effectiveness and better safety outcomes.
▪ TactiFlex SE’s flexible tip was more stable than solid catheters; this improved stability and resulted in more efficient procedures.

## Introduction

Atrial fibrillation (AF) is a leading public health concern, as it is closely associated with heart failure, stroke, cardiovascular mortality, decreased quality of life, and increased healthcare burden.^1^^,^^2^ Atrial flutter (AFL) often accompanies AF, and the prevalence of individuals with AF/AFL is estimated at >37.65 million worldwide.^1^^,^^2^ One in 4 middle-aged adults are predicted to develop these arrhythmias in their lifetime.^2^

Management of AF includes treatment with rate-control measures, antiarrhythmic drugs (AADs), and interventional procedures, typically isolation of the pulmonary veins (PVs) by ablation.^2^^,^^3^ A Class I recommendation by the 2017 Heart Rhythm Society/European Heart Rhythm Association/European Cardiac Arrhythmia Society/Asia Pacific Heart Rhythm Society/Sociedad Latinoamericana de Estimulación Cardíaca y Electrofisiología expert consensus statement, ablation improves long-term clinical outcomes with relatively low risk.^3^ Strides have been made to improve the safety, efficiency, and efficacy of ablation, including usage of cardiac imaging, 3-dimensional mapping systems, open-irrigation catheters, and real-time feedback of contact force (CF).^3^, ^4^, ^5^ Improved procedural efficiency has been associated with improved long-term clinical success.^4^^,^^6^, ^7^, ^8^

The TactiFlex Ablation Catheter, Sensor Enabled (TactiFlex SE) (Abbott, Abbott Park, IL) is the first CF-sensing radiofrequency (RF) ablation catheter with a flexible tip. Proven white light interferometry principles with fiberoptic technology allow for accurate measurement of CF.^9^ A porous flexible distal tip design allows for saline irrigation through kerfs laser cut in an interlocking pattern. Two magnetic sensors and updated software allow for precise visual localization and display of a real-time CF arrow and deflection direction indicators at the catheter’s tip when used with the EnSite X EP System.

This study was conducted to evaluate the safety and effectiveness of TactiFlex SE for the treatment of drug-refractory, symptomatic paroxysmal AF (PAF). Secondary objectives were to provide supporting data on safety and effectiveness (1) at 40 to 50 W in the left atrium (LA) and (2) for right atrial ablation of cavotricuspid isthmus (CTI)–dependent (or typical) AFL.

## Methods

### Study population

This was a prospective, nonrandomized, multicenter clinical trial to evaluate the safety and effectiveness of ablation with the TactiFlex SE catheter for the treatment of PAF compared with predetermined performance goals. This clinical study was conducted under an investigational device exemption (IDE). The study design included a main study cohort in which operators could choose their ablation power settings from within the recommended range of 20 to 50 W and a high standard power (HSP) substudy in which operators were required to use ablation power settings of 40 to 50 W in the LA. A total of 355 subjects were enrolled in this study worldwide, 305 in the main study and 50 in the HSP substudy, at 37 clinical sites in the United States, Europe, Australia, Hong Kong, and Taiwan. Enrollment began on June 26, 2020, and concluded on June 18, 2021. No site could contribute more than 20% of the total enrollments. Every participant provided written informed consent, and the study was approved by the Institutional Review Board or Ethics Committee at all participating institutions. This clinical investigation was conducted in accordance with the Declaration of Helsinki.

Subjects were ≥18 years of age and undergoing a planned ablation procedure for symptomatic drug-refractory PAF. Subjects had to have 1 electrocardiographically documented AF episode within 12 months prior to informed consent/enrollment and a physician’s note indicating recurrent self-terminating AF. Key exclusion criteria were persistent AF, LA diameter >5.0 cm, left ventricular ejection fraction <35%, New York Heart Association functional class III or IV, body mass index >40 kg/m^2^, and previous ablation therapy in the LA. Table 1 summarizes the baseline characteristics. Baseline quality-of-life assessments included administration of the AF Effect on Quality-of-Life Questionnaire (AFEQT) and EQ-5D-5 questionnaires.

**Table 1:** Baseline demographics of all treated subjects (N = 334)

Characteristic	All treated subjects
Age, y	65.0 (56.0–71.0)
Male	214 (64.1)
Body mass index, kg/m^2^	28.4 (25.7–32.8)
CHA_2_DS_2_-VASc Score	2.0 (1.0–3.0)
Left ventricular ejection fraction, %	60.0 (55.0–60.0)
Left atrial diameter, mm	40.0 (36.0–43.0)
Atrial flutter (typical)	47 (14.1)
Atrial flutter (atypical)	4 (1.2)
Atrial flutter (unknown)	31 (9.3)
Atrial tachycardia	9 (2.7)
Ventricular tachycardia	24 (7.2)
First-degree heart block	10 (3.0)
Second-degree heart block	1 (0.3)
Third-degree heart block	1 (0.3)
AV nodal dysfunction	3 (0.9)
Supraventricular tachycardia	1 (0.3)
Pacemaker or implantable cardiac monitor	22 (6.6)
Coronary artery disease	56 (16.8)
Coronary artery bypass graft surgery	4 (1.2)
Diabetes	41 (12.3)
Heart Failure	71 (21.3)
Hypertension	197 (59.0)
Myocardial infarction	15 (4.5)
Obstructive sleep apnea	62 (18.6)
Stroke	12 (3.6)
Transient ischemic attack	5 (1.5)
Structural heart disease	21 (6.3)
History of alcohol abuse	5 (1.5)

Values are median (interquartile range) or n (%).

CHA_2_DS_2_-VASc = congestive heart failure, hypertension, age ≥75 years, diabetes mellitus, prior stroke or transient ischemic attack or thromboembolism, vascular disease, age 65-74 years, sex category.

### Ablation procedure

A strategy of uninterrupted oral anticoagulation was recommended for all ablation procedures, and all patients were required to undergo screening for LA thrombus within 1 day of the procedure. All ablation procedures were performed with the TactiFlex SE catheter and included PV isolation (PVI) followed by a minimum 20-minute wait period to confirm electrical isolation of each PV via entrance block. If PV reconnection occurred during or after the 20-minute waiting period, additional lesions were administered. Posterior wall isolation or additional ablation lines were allowed but were not part of the recommended ablation strategy. Subjects with a history of typical AFL or induced CTI-dependent AFL were required to undergo concomitant AFL ablation, which was confirmed by bidirectional block. In the main study cohort, operators could choose their catheter settings from within recommended ablation parameters (20–50 W, 5–20 g CF, 13 mL/min irrigation flow rate during ablation). In the HSP substudy, operators were limited to 40 to 50 W for LA ablations. Operators were free to choose their ablation technique (point by point, drag) and method of assessing RF lesion quality (impedance drop, time, etc.). Esophageal temperature monitoring was strongly encouraged, and discontinuation of ablation energy for any esophageal temperature rise of >1°C was recommended. Before discharge, patients underwent physical examination and National Institute of Health Stroke Scale assessment.

### Postablation monitoring and follow-up

Scheduled follow-up occurred at 7 days, 5 weeks, 3 months, 6 months, and 12 months after ablation. Consistent with recommendations, after ablation there was a 90-day blanking period.^3^ One repeat ablation procedure was allowed in the blanking period. At 5 weeks, subjects were reminded to discontinue AADs unless clinically justified. Quality-of-life questionnaires were administered at the 3-month, 6-month, and 12-month visits. A 12-lead electrocardiogram was performed at the 3- and 12-month visits. At least 1 transtelephonic transmission was collected in every 14-day period between the 3- and 12-month visits. At 12 months, a 24-hour Holter monitor was administered.

### Outcomes

Two primary endpoints, 3 secondary endpoints, and several descriptive endpoints were prospectively collected. The primary safety endpoint was the rate of device and/or procedure-related serious adverse events with onset within 7 days of any ablation procedure that used the TactiFlex SE catheter. Predefined primary safety events (PSEs) are shown in Table 2. Relatedness of the event to the catheter and/or procedure was determined by the Clinical Events Committee (CEC). Data for the periprocedural complication rate observed in previous trials ranged from 2.5% to 8.9% with an average of 6.47%.^9^, ^10^, ^11^, ^12^, ^13^, ^14^, ^15^ In consultation with the Food and Drug Administration, the performance goal for the primary safety endpoint was calculated to be 2 times the expected event rate: 2 × 6.47%, which is equal to 12.9%.

**Table 2:** Primary safety endpoint events (safety population) (N = 330)

Endpoint criteria	Number of events	Proportion of subjects (%)
Vascular access complications (including major bleeding events)	5	1.5
Cardiac tamponade/perforation	3	0.9
Pericarditis	2	0.6
Pulmonary edema	2	0.6
Transient ischemic attack	1	0.3
Stroke/cerebrovascular accident	1	0.3
Atrio-esophageal fistula	0	0.0
Death	0	0.0
Heart block	0	0.0
Myocardial infarction	0	0.0
Phrenic nerve injury resulting in diaphragmatic paralysis	0	0.0
Pulmonary vein stenosis	0	0.0
Thromboembolism	0	0.0
Vagal nerve injury/gastroparesis	0	0.0
Total	14	4.2

There was 1 occurrence of esophageal pericardial fistula that did not meet the prespecified definition for a primary safety endpoint event. After surgical intervention, this subject made a full recovery.

The primary effectiveness endpoint was freedom from documented AF/AFL/atrial tachycardia (AT) episodes of >30 seconds duration that were documented by 12-lead electrocardiography, transtelephonic transmission, or Holter monitor after the 90-day blanking period through 12 months of follow-up. Primary effectiveness endpoint failures also included failure to achieve acute procedural success, an ablation in the LA using an ablation catheter other than the TactiFlex SE, a repeat ablation procedure >80 days after the initial procedure, cardioversion after the blanking period, or use of a new AAD or an AAD at a dose higher than a historical maximum dose.

The symptomatic secondary effectiveness endpoint had the same definition as the primary effectiveness endpoint, except that documented recurrence without documented evidence of symptoms after the 90-day blanking period did not count as a failure in this analysis. The single-procedure secondary effectiveness endpoint had the same definition as the primary effectiveness endpoint, except that any repeat ablation in the LA counted as a failure in this analysis. The AAD-free secondary effectiveness endpoint had the same definition as the primary effectiveness endpoint, except that any use of Class I or III AADs after the 90-day blanking period counted as a failure in this analysis.

### Statistical methods

The primary safety endpoint performance goal was achieved if the upper bound of the 1-sided 97.5% exact binomial confidence limit for the proportion of subjects with a primary safety endpoint was below 12.9% using Fisher’s exact test. The study sample size was determined by the primary safety endpoint.

The primary effectiveness endpoint rate at 12 months was calculated using the Kaplan-Meier (KM) method. The performance goal was achieved if the lower bound of the 1-sided 97.5% KM survival estimate confidence interval (CI) was >50%, which was set based on the chronic acceptable success rate for paroxysmal AF at 12-months.^3^ The Food and Drug Administration provided feedback that a performance goal of 50% was acceptable if the primary effectiveness endpoint was based on any documented AF/AFL/AT recurrence.

If the primary safety and primary efficacy endpoints were both met, the secondary endpoints could be tested in the specified hierarchical order to control for type I error. The symptomatic secondary effectiveness endpoint performance goal was achieved if the lower bound of the 97.5% KM survival estimate CI was <55%. The single-procedure secondary effectiveness and AAD-free secondary effectiveness endpoint performance goals were achieved if the lower bound of the 97.5% KM survival estimate CI was >50%.

Patient demographics and additional data were summarized descriptively. Continuous variables were summarized with median and interquartile range (IQR) and number of observations. Categorical variables were summarized as count and percentage. Procedural characteristics were evaluated using a 2-sided *t* test. Fisher’s exact test was used to compare proportions between groups experiencing vascular access complications. AFEQT assessments compared the proportion of subjects experiencing AF in follow-up visits to the proportion at baseline using McNemar’s chi-square test for paired data. AFEQT overall scores and the EQ visual analog scale (EQ-VAS) scores at follow-up visits were compared with baseline using the Wilcoxon signed rank test.

Methods and subject baseline characteristics from the TactiCath Contact Force Ablation Catheter, Sensor Enabled IDE have been previous described.^9^

Statistical analyses were performed with the use of SAS software version 9.4 (SAS Institute, Cary, NC). The trial is registered with ClinicalTrials.gov (NCT04356040).

## Results

**Figure 1:**
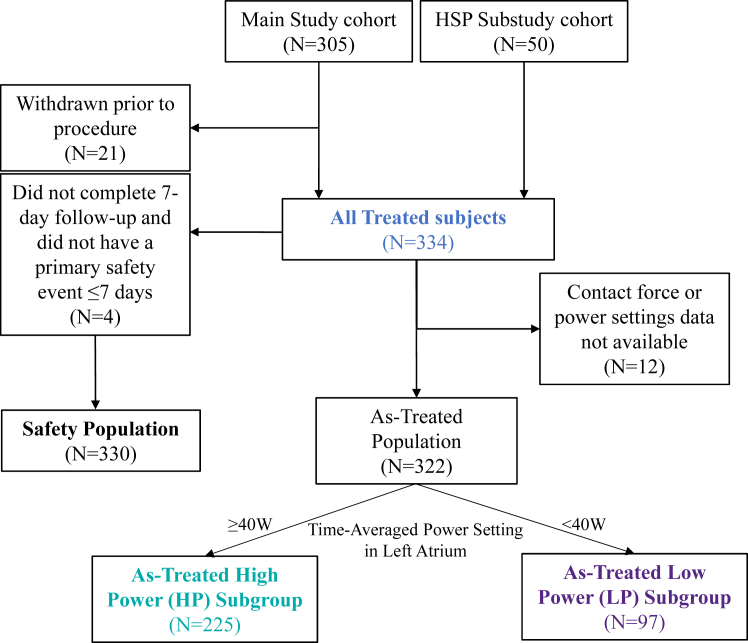
Subject disposition for analysis populations. Definition of the safety, all-treated, and as-treated populations. HSP = high standard power.

A total of 355 subjects were enrolled at 37 clinical sites worldwide from June 26, 2020, to June 18, 2021. Included in the primary safety analysis population were 330 subjects who had the investigational catheter inserted and either completed their 7-day visit or had a primary safety endpoint event within 7 days of the procedure (Figure 1). All treated subjects (N = 334) included all subjects who had the investigational catheter inserted into their vasculature and RF energy delivered. The as-treated population (n = 322) included all treated subjects in which CF and power setting data was available. In the as-treated population, 225 subjects had a time-averaged power setting of ≥40 W (high power [HP]) and 97 had a time-averaged power setting <40 W (low power [LP]). Overall, 93.4% (n = 243 of 334) of treated subjects completed 12 months of follow-up.

Baseline characteristics are summarized in Table 1. Of the 334 treated subjects, 214 (64.1%) were male, and the overall median age was 65 years (IQR 56–71 years). The most common comorbidity was hypertension (59.0%). Additionally, 14.1% of treated subjects had a history of typical AFL.

### Procedural data

Procedural data are shown in Table 3 for all treated subjects as well as for the HP and LP subjects. The median total procedure time for all subjects was 119.5 minutes, with a median total RF time of 20.0 minutes, a median total RF time for PVI of 17.0 minutes, a median total fluoroscopy time of 5.0 minutes, and a median total irrigation fluid volume of 436.0 mL. Non-PV ablations beyond PVI were performed in 43.4% (n = 145 of 334) of all subjects and are summarized in Supplemental Table 1. The most common non-PV ablation strategy was a CTI line for typical AFL in 33.5% (n = 112 of 334) subjects. Esophageal monitoring was performed in 72.8% (n = 243 of 334) of procedures and resulted in RF interruption in 37 cases.

**Table 3:** Procedural data

Characteristic	HP subjects (n = 225)	LP subjects (n = 97)	*P* value∗	All treated subjects (N = 334)
Total procedure time, min	111.0 (92.0–134.0)	151.0 (122.0–191.0)	<.0001	119.5 (98.0–152.0)
Total PV ablation time, min	43.0 (30.0–65.0)	78.0 (56.0–100.0)	<.0001	50.5 (33.0–76.0)
Total RF time, min	17.0 (12.0–22.5)	36.0 (26.0–46.0)	<.0001	20.0 (14.0–30.0)
Total RF time for PV ablation, min	14.0 (11.0–19.0)	32.5 (25.0–41.5)	<.0001	17.0 (13.0–26.5)
Total fluoroscopy time, min	4.0 (0.0–11.0)	10.0 (5.0–18.0)	<.0001	5.0 (0.0–13.0)
Total irrigation fluid volume, mL	378.0 (310.0–486.0)	691.5 (515.5–891.0)	<.0001	436.0 (336.0–593.0)

Values are median (interquartile range).

HP = high power; LP = low power; PV = pulmonary vein; RF = radiofrequency.

∗ Two-sided t-test. All *P* values are displayed for information purposes only; they were not from prespecified hypothesis testing.

**Figure 2:**
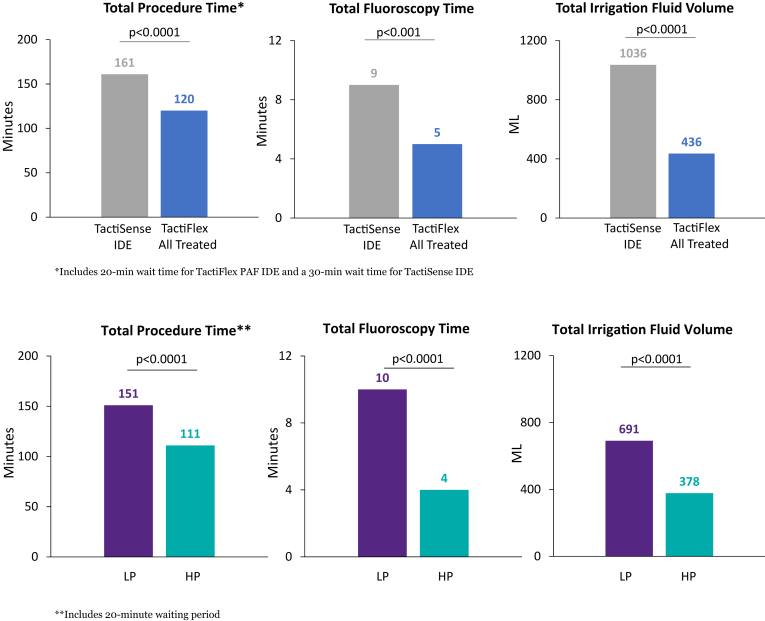
Procedural data. Procedural data results from the TactiFlex AF paroxysmal atrial fibrillation (PAF) (TactiFlex SE, all treated subjects [N = 334]) and TactiSense investigational device exemption (IDE) (TactiCath SE, all treated subjects [N = 151]) studies. The TactiSense IDE was the PAF pivotal study for TactiCath SE. HP = high power; LP = low power.

A comparison of procedural data from this study with the TactiSense IDE trial (TactiCath SE) demonstrates improved clinical efficiency with the TactiFlex SE catheter vs the TactiCath SE catheter (Figure 2). With the TactiFlex SE, there were significant reductions in procedure time (120 [IQR 98–152] minutes vs 161 [IQR 123–206] minutes; *P* < .0001), total fluoroscopy time (5.0 [IQR 0–13] minutes vs 9.0 [IQR 5–17] minutes; *P* < .001), and total irrigation fluid volume (436 [IQR 336–593] mL vs 1036 [IQR 838–1450] mL; *P* < .0001). Comparing procedural data from the HP and LP groups demonstrates a significant decrease in the HP group for procedure time (111 [IQR 92–134] minutes vs 151 [IQR 122–191] minutes; *P* < .0001), total fluoroscopy time (4.0 [IQR 0–11] minutes vs 10.0 [IQR 5–18] minutes; *P* < .0001), and total irrigation fluid volume (378 [IQR 310–486] mL vs 692 [IQR 516–891] mL; *P* < .0001).

### Safety assessments

There were 14 PSEs in 14 of 330 subjects, an observed rate of 4.2% (Table 2). The 97.5% CI upper bound was 7%, which was below the predetermined performance goal of 12.9%, and the primary safety endpoint was met. The most common complication was related to vascular access, which occurred in 5 subjects. As recommended in the protocol to minimize complications, ultrasound was used to obtain vascular access in 219 of 333 subjects. Vascular access complications (both serious and nonserious) were significantly lower in procedures that utilized ultrasound to obtain access (0.9%) compared with procedures that did not use ultrasound to obtain access (6.1%) (*P* = .0089). The proportion of LP subjects with a PSE was 5.2% (n = 5 of 97) compared with 4.1% (n = 9 of 222) for HP subjects (*P* = .7671).

Three cases of cardiac tamponade/perforation occurred. All cases were resolved, 2 with percutaneous intervention and the other with surgical intervention. One subject experienced an esophageal pericardial fistula and fully recovered after surgical intervention. Review of the EnSite case file revealed that a small region (∼6 mm) near the left inferior PV received >50 seconds of RF energy at 50 W and up to 30 g CF, 5 times the maximum duration recommendation of 10 seconds and twice the maximum recommendation (<15 g) for CF on the posterior wall in the instructions for use (IFU).

Two deaths occurred in the study. Both were adjudicated by the CEC as resulting from noncardiovascular causes (motorcycle crash, infection) with 1 of the deaths (infection) adjudicated as potentially related to the index study procedure but not meeting primary safety endpoint criteria. This subject was readmitted to the hospital on postoperative day 23 and developed an infection on day 27. Three days later the subject’s condition remained poor, and the family decided to withdraw care to comfort only. The subject died the following day. The CEC review of all hospital records and test results specifically investigating a potential esophageal fistula event remained inconclusive and led to the adjudication. Review of the EnSite case file revealed several areas where both RF duration (at 50 W) and CF exceeded IFU guidelines.

### Effectiveness assessments

First-pass isolation, defined as confirmation of entrance block in all PVs following the initial minimum waiting period of 20 minutes during the initial ablation procedure was achieved in 80.5% (n = 269 of 334) subjects. The HP subjects experienced a higher rate of first-pass isolation than LP subjects (81.8% and 77.3%, respectively). Acute procedural success was achieved in 99.4% (n = 332 of 334) subjects.

**Figure 3:**
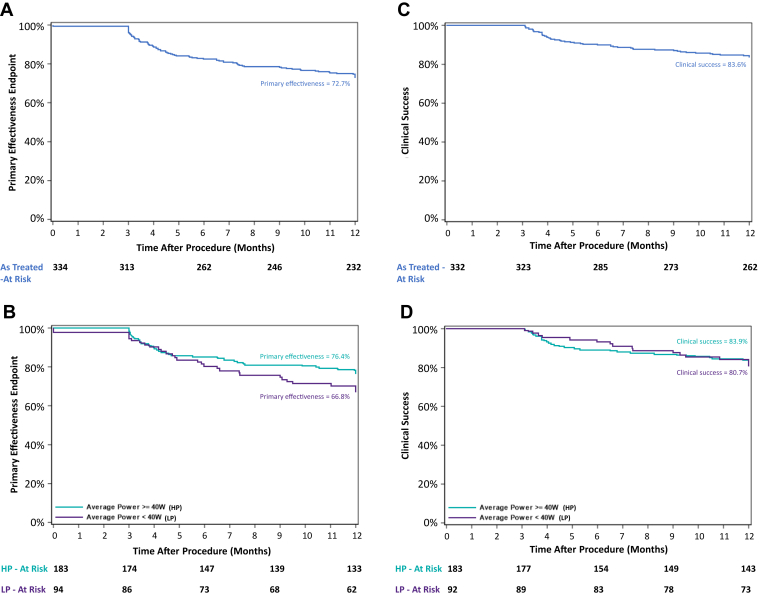
Primary effectiveness endpoint and clinical success. Kaplan-Meier estimates of primary effectiveness in the all-treated population (A) and high-power (HP) and low-power (LP) populations (B); and clinical success in the all-treated population (C) and HP and LP populations (D).

The primary effectiveness endpoint (12 months) was achieved in 72.7% of all treated subjects (Figure 3A). The 1-sided 97.5% CI lower bound was 67.5%, greater than the predefined performance goal of 50%; thus, the primary effectiveness endpoint was met. A total of 87 (26.0%) of 334 subjects failed 1 or more endpoint criteria, the most common being documented recurrence of AF, AFL, and/or AT (85.1% [n = 74 of 87]). Two subjects failed to achieve acute procedural success but did not meet any other failure criteria. The primary effectiveness endpoint was also evaluated for the as-treated populations and was achieved in 76.4% (95% CI 69.3%–82.0%) of the HP population compared with 66.8% (95% CI 56.1%–75.5%) in the LP population (Figure 3B).

The symptomatic secondary effectiveness endpoint was achieved in 79.7% of subjects. The 1-sided 97.5% KM lower bound was 74.9%, which is greater than the predefined performance goal of 55.0%. The symptomatic effectiveness endpoint was achieved in 75.4% of LP subjects and 81.5% of HP subjects (Table 4). The single procedure secondary effectiveness endpoint was achieved in 71.5% of subjects. The 97.5% KM lower bound was 66.2%, which is greater than the predefined performance goal of 50%. The single procedure secondary effectiveness endpoint was achieved in 65.8% of LP subjects and 74.5% of HP subjects. AAD-free effectiveness was achieved in 64.3% of subjects. The 97.5% KM lower bound was 58.8%, which is greater than the predefined performance goal of 50.0%. The secondary AAD-free effectiveness endpoint was achieved in 59.4% of LP subjects compared with 67.4% of HP subjects.

**Table 4:** Secondary endpoints

Endpoint	Rate (%)	97.5% KM lower bound (%)	Performance goal (%)
Symptomatic effectiveness			
All treated	79.7	74.9	>55.0
LP	75.4	65.3	N/A
HP	81.5	75.7	N/A
Single-procedure effectiveness			
All treated	71.5	66.2	>50.0
LP	65.8	55.2	N/A
HP	74.5	68.2	N/A
AAD-free effectiveness			
All treated	64.3	58.8	>50.0
LP	59.4	48.7	N/A
HP	67.4	60.7	N/A

AAD = antiarrhythmic drug; HP = high power; KM = Kaplan-Meier; LP = low power; N/A = not applicable.

The KM estimate of clinical success (defined as the secondary symptomatic effectiveness endpoint rate after excluding acute procedural success failure subjects and not counting AAD-use as an endpoint failure) was 83.6% (95% CI 78.1%–87.2%) in all treated subjects (Figure 3C). Clinical success was achieved in 80.7% (95% CI 70.8%–87.5%) of LP subjects and 83.9% (95% CI 77.5%–88.6%) of HP subjects (Figure 3D).

The proportion of subjects who experienced at least 1 repeat ablation for AF/AFL/AT during the study was 6.0% (n = 20 of 334); these results are summarized in Supplemental Table 2. Excluding procedures to treat CTI-dependent (typical) flutter, the proportion of subjects who experienced at least 1 repeat ablation during the study was 5.1% (n = 17 of 334). There were 8 repeat procedures during the blanking period and an additional 12 procedures through 12 months. Two additional subjects experienced their only repeat procedure after 12 months, but prior to their last study visit (the total number of repeat procedures anytime during study period was 22).

### Quality of life

AFEQT scores improved from 61.9 at baseline to 84.7 at 3 months (*P* < .01) and further increased to 87.2 at 12 months (*P* < .01). The proportion of subjects reporting an episode of AF in the previous month dropped significantly (*P* < .01) from 64.0% (n = 213 of 333) at baseline to 12.2% (n = 38 of 311) at 12 months.

The EQ-5D-5L evaluation also showed notable improvement from baseline to 12-month follow-up, particularly with decreases in moderate-to-severe issues in the anxiety/depression dimension from baseline (12.9%) through 12 months (6.43%) (*P* < .01), and decreases in moderate-to-severe issues reported for usual activities from baseline to 6 months (10.8% to 4.44%; *P* < .01) and 12 months (10.8% to 6.75%; *P* < .05).

Significant improvements were seen at 12 months in the EQ-VAS, which asks subjects to rate their health on a vertical line labeled 0 to 100. At baseline, the mean EQ-VAS score was 76.05 ± 15.40, and at 12 months the mean had increased to 83.18 ± 13.45 (*P* < .01).

## Discussion

This study demonstrated the use of the TactiFlex SE ablation catheter at ablation power settings up to 50 W is safe and effective for the treatment of symptomatic, drug-refractory PAF and concomitant typical AFL. Based on the prespecified margins, the TactiFlex SE achieved the performance goals for both safety and effectiveness.

The absence of a control group is a limitation of the study. To place these results in context, results can be compared with similar studies, including the IDE study for the previous generation device (TactiCath SE).^9^ The 12-month primary effectiveness endpoint was achieved in 72.7% of all treated subjects, comparable to historical studies such as the SMART AF trial (72.5%, THERMOCOOL® SMARTTOUCH™ Catheter for the Treatment of Symptomatic Paroxysmal Atrial Fibrillation).^6^^,^^8^^,^^9^ TactiFlex PAF IDE utilized stringent arrhythmia recurrence monitoring throughout the follow-up period (3–12 months) for evaluation of its effectiveness endpoints.

The PSE rate was 4.2%, comparable to similar trials in PAF populations.^5^^,^^6^^,^^8^^,^^9^^,^^16^ No study subjects experienced atrioesophageal fistulas, PV stenosis, heart block, myocardial infarction, phrenic nerve injury resulting in diaphragmatic paralysis, thromboembolism, cardiovascular-related death, or vagal nerve injury/gastroparesis. In the 2 most serious events related/likely related to the procedure, examination of the EnSite case files revealed that IFU recommendations for duration and CF were greatly exceeded, reinforcing the importance of IFU guidelines, particularly in more vulnerable regions such as the posterior wall.

The rate of procedure-related vascular access complications was significantly lower in subjects in whom ultrasound was used to obtain vascular access compared with subjects in whom ultrasound was not used. Additionally, vascular access complications, including major bleeding events, were responsible for 5 of the 14 total PSEs that occurred in the study. This suggests that usage of ultrasound to obtain vascular access may reduce procedure-related complications and should be strongly recommended in ablation procedures.^5^

Ablation at higher power settings (≥40 W) is a topic of interest, as it may yield better efficacy in AF patients, in addition to efficiencies such as reduced procedure time, limited patient exposure to fluoroscopy radiation, and a reduction in irrigation fluid. This study predefined an as-treated subanalysis using time-averaged power settings to define HP (40–50 W) vs LP (<40 W) subgroups. This study was not powered to compare endpoints between the HP and LP groups. The HP group experienced a lower rate of PSEs at 90 days (4.1%) compared with the LP group (5.2%) as well as a higher rate of first-pass isolation compared with the LP group (81.8% and 77.3%, respectively). The HP group outperformed the LP group in other procedural efficiencies including a 57% reduction in median RF time for PV ablation, 26% reduction in total procedure time, 60% reduction in total fluoroscopy time, and a 45% reduction in total irrigation fluid volume. At 12 months, HP showed favorable symptomatic effectiveness (HP 81.5% and LP 75.4%) and primary effectiveness rate (HP 75.5% and LP 67.9%). Single procedure effectiveness post–blanking period was also favorable for HP than LP (HP 74.5% and LP 65.8%). These results re-emphasize that the TactiFlex SE is safe and effective for use in the treatment of paroxysmal AF in the full range of 20 to 50 W. Usage of the TactiFlex SE at power ranges ≥40 W may be more procedurally efficient and clinically effective than usage at lower powers. Previous meta-analyses have concluded that there is no difference in the safety risk profile between high power, short duration (HPSD) (≥40 W) and low power, long duration (<40 W),^17^^,^^18^ and that HPSD may have a lower risk of esophageal thermal injury.^19^ However, a recent randomized trial observed higher rates of asymptomatic cerebral emboli in the HPSD arm,^20^ and if this observation is replicated additional study on the significance of these asymptomatic cerebral emboli may be warranted.

Additional comparisons can be made between TactiFlex PAF IDE’s HP group (40–50 W) and Biosense Webster’s Q-FFICIENCY (Evaluation of QDOT MICRO™ Catheter for Pulmonary Vein Isolation in Subjects With Paroxysmal Atrial Fibrillation) IDE trial’s very high power short-duration (vHPSD) group (90 W, 4 sec). Q-FFICIENCY was a prospective nonrandomized premarket clinical trial to evaluate the safety and efficacy of the QDOT MICRO Catheter in PAF subjects ablated at 90 W for ≤4 seconds for PVI.^6^ The Q-FFICIENCY (all treated subjects N = 164) and TactiFlex PAF IDE (all treated subjects N = 334; HP, n = 225) trials had comparable acute procedural success (100% for the Q-FFICIENCY trial and the TactiFlex PAF IDE trial HP group; 99.4% for the TactiFlex PAF IDE trial all subjects; 100% for the TactiFlex PAF IDE trial HP group). TactiFlex PAF IDE achieved a higher first-pass isolation rate (80.5% all treated subjects, 81.8% HP group) compared with Q-FFICIENCY (67.4% when vHPSD-only mode was used for PVI). Despite the Q-FFICIENCY trial having a lower total RF time (median 9.8 minutes) than the TactiFlex PAF IDE trial (median 20 minutes, all subjects [n =333]), both studies had similar median total ablation times for their catheters (QDOT at 65.0 minutes, TactiFlex SE at 69.5 minutes), and the TactiFlex PAF IDE trial had a lower median total procedure time (132.0 minutes in the Q-FFICIENCY trial; 119.5 minutes in the TactiFlex PAF IDE trial all subjects; 111.0 minutes in the TactiFlex PAF IDE trial HP group).^6^ KM 12-month primary effectiveness rate was 76.4% in the TactiFlex PAF IDE trial HP subjects, comparable to 76.7% observed in the Q-FICCIENCY trial. Additionally, clinical success was achieved in 83.9% TactiFlex PAF IDE HP subjects, similar to that observed in the Q-FICCIENCY trial (86.0%).^6^ Thus, the TactiFlex SE catheter at power settings ≤50 W performs comparably to the QDOT MICRO catheter at its highest power setting of 90 W. These results are consistent with other findings that ablation at HP yields better acute results and comparable long-term outcomes as vHPSD group ablation.^7^ AF ablation at 40 to 50 W, or HP, may provide the best of all worlds, combining greater procedural efficiency and accuracy with favorable long-term outcomes.

**Figure 4:**
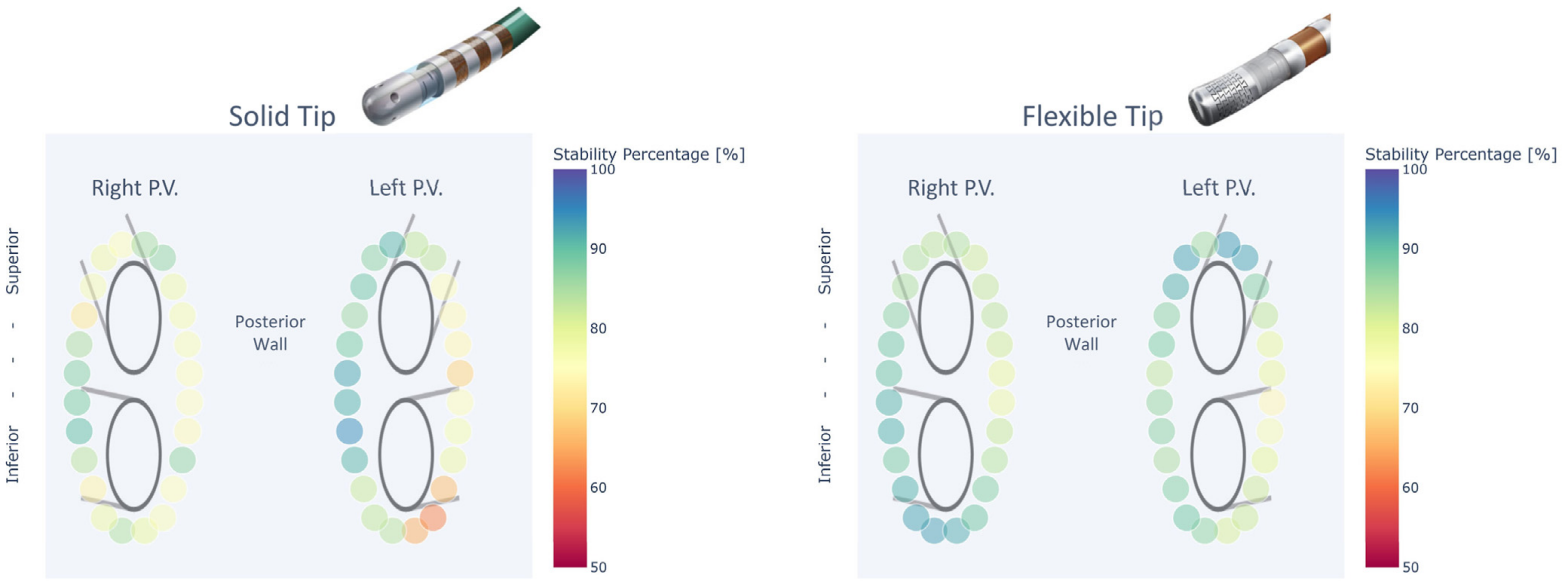
Catheter stability. Stability of the solid tip (TactiCath SE) (left) and flexible tip (TactiFlex SE) (right) catheters related to the left atrial pulmonary vein (PV) anatomy. Each ablation lesion marker is associated with the group of radiofrequency episodes in the respective anatomic region and represents the mean percentage of time during an radiofrequency episode where the ablation electrode remained within 2.5 mm of the ablation lesion centroid.

Preclinical bench testing comparing the TactiFlex SE with its previous generation catheters, the TactiCath SE and FlexAbility SE, showed that both catheters generate lesions of comparable width during RF application.^21^^,^^22^ Bench testing also exhibited that flexible-tip catheters, such as the TactiFlex SE, required a greater displacement force at CF >5 g when compared with conventional smooth tip catheters, such as the TactiCath SE. These findings suggest that greater stability may be observed when operating flexible-tip catheters, which may lead to greater predictability of lesion size.^23^ Additionally, a retrospective study compared the catheter stability of a flexible-tip catheter (TactiFlex SE) to a solid-tip catheter (TactiCath SE) during RF applications within the TactiFlex AF IDE and the TactiSense IDE trials. When PVI and CTI lesions from point-by-point ablation style operators with discrete lesions were evaluated, the TactiFlex SE had superior positional stability when compared with the TactiCath SE. Increased stability with flexible-tip catheters over solid-tip catheters has been shown regardless of anatomic location around the PV ostium (Figure 4).^23^^,^^24^ Thus, the TactiFlex SE offers an advantage over solid-tip ablation catheters including its predecessor the TactiCath SE. This may lead to favorable outcomes when TactiFlex SE is used for ablation as stability is a significant factor in lesion durability and long-term AF ablation effectiveness.^4^

## Conclusion

The TactiFlex PAF IDE trial demonstrated that the TactiFlex SE catheter is safe and effective for the treatment of drug-refractory symptomatic PAF and concomitant typical AFL, reducing arrhythmia recurrence while improving quality of life. This is the first catheter to provide both the benefits of CF sensing and the stability of a flexible tip, resulting in more efficient ablation procedures.
