# Supplementary material for: Safety and effectiveness of the first contact force ablation catheter with a flexible tip
Source: Heart Rhythm O2. 2023 Oct 31;4(12):784–93. doi: 10.1016/j.hroo.2023.10.006 (PMC10774658; doi:10.1016/j.hroo.2023.10.006)
Supplement: Supplemental Tables [file mmc1.docx]

Supplementary Table 1. Non-Pulmonary Vein Ablations

| **Non-PV Ablation Strategy** | **All Treated Subjects**  **(N=334)** |
| --- | --- |
| Typical Atrial Flutter | 112 (33.5%) |
| Linear Lesion | 31 (9.3%) |
| Roof Line | 25 (7.5%) |
| Posterior Line | 25 (7.5%) |
| SVT | 12 (3.6%) |
| CFAE Sites | 9 (2.7%) |
| Atypical Atrial Flutter | 9 (2.7%) |
| Mitral Isthmus | 6 (1.8%) |
| Atrial Tachycardia | 4 (1.2%) |
| Superior Vena Cava | 4 (1.2%) |
| Coronary Sinus | 1 (0.3%) |
| Rotational Activity | 1 (0.3%) |
| Scar Ablation | 1 (0.3%) |
| Other | 1 (0.3%) |

Supplementary Table 2. Repeat Ablation Details

| Description | HP Subjects  (N=225) | LP Subjects  (N=97) | All Treated Subjects (N=334) |
| --- | --- | --- | --- |
| Subjects with repeat procedure for AF/AFL/AT recurrence | 13 (5.8%) | 7 (7.2%) | 20 (6.0%) |
| Number of repeat procedures for AF/AFL/AT recurrence^1^ | 13 | 9 | 22 |
| Number of repeat procedures during blanking period (≤90 days) | 3 | 5 | 8 |
| Days since initial procedure (mean ± SD) | 45.0 ± 7.0 | 65.6 ± 6.6 | 57.9 ± 16.8 |
| Reason for repeat procedure |  |  |  |
| AF/AT/Atypical Flutter | 2 | 4 | 6 |
| CTI-Dependent (Typical) Flutter | 1 | 1 | 2 |
| Number of repeat procedures after blanking period through 12-months^2^ | 9 | 3 | 12 |
| Days since initial procedure (mean ± SD) | 210.4 ± 28.6 | 252.0 ± 28.0 | 220.8 ± 78.4 |
| Reason for repeat procedure |  |  |  |
| AF/AT/Atypical Flutter | 8 | 2 | 10 |
| CTI-Dependent (Typical) Flutter | 1 | 1 | 2 |

^1^Two subjects experienced two repeat procedures, one during the blanking period and one later in the study (day 266 and 292).

^2^Two subjects experienced their only repeat procedure after day 365 but prior to their last study visit.
